# Supplementary material for: α‐Synuclein oligomers slow down action potential firing and enhance dopamine release by increasing Cav2.2 currents in midbrain dopaminergic neurons
Source: J Physiol. 2026 Mar 16;604(7):3094–113. doi: 10.1113/JP288914 (PMC13039254; doi:10.1113/JP288914)
Supplement: Supplementary file 2 — Statistical Summary [file TJP-604-3094-s002.docx]

| **FIGURE 1** |
| --- |

|  | Ctrl | *a-syn* | Test statistic | p |
| --- | --- | --- | --- | --- |
| (% of Tuj1 on DAPI | 6.30±0.97  n=17834 | 6.10±0.93  n=25173 | Mann-Whitney | 0.342 |

| **FIGURE 2** |
| --- |

|  | Ctrl | *a-syn* | Test statistic | p | DIV |
| --- | --- | --- | --- | --- | --- |
| Spontaneous firing discharge | 4.6±3.6Hz n=38 | 1.9±2.1 Hz n=50 | KW-ANOVA | 0.0012 (***) | 11 DIV |
| Interevent interval | 0.43±0.42 s n=37 | 0.86±0.68 s n=40*;* | Mann-Whitney Test | 0.001 (***) | 11 DIV |
| Rm | 0.47±0.11 GΩ n=27 | 0.42±0.0.11GΩ  n=33 | Mann-Whitney Test | 0.375 | 11 DIV |

| **FIGURE 3** |
| --- |

|  | Ctrl | *a-syn* | Test statistic | p | DIV |
| --- | --- | --- | --- | --- | --- |
| Half-width | 3.7±1.18 ms n=37 | 3.2±1.34 ms n=41 | Mann-Whitney Test | 0.04 (*) | 11 DIV |
| Tp | 6.0±0.4 ms n=37 | 5.8±0.3 ms n=41*;* | Mann-Whitney Test | 0.001 (***) | 11 DIV |
| AHP | -44.3±5 mV n=37 | -46.7±4.42 mV  N=41 | t-test | 0.009 (**) | 11 DIV |
| dV/dtmax | 107±48 mV/ms n=46 | 159±44 mV/ms  n=30 | One-way ANOVA | 0.0001 (***) | 11 DIV |
| overshoot | 38.1±10.3 mV n=37 | 41.8±10.7 mV  n=41 | Mann-Whitney Test | 0.211 | 11 DIV |
| threshold | -39.4±3.8 mV n=37 | -41.9±4.5 mV  n=41 | Mann-Whitney Test | 0.038 (*) | 11 DIV |

| **FIGURE 4** |
| --- |

|  | Ctrl | *a-syn* | Test statistic | p | DIV |
| --- | --- | --- | --- | --- | --- |
| dV/dtmax | 75.34±17.76 mV/ms n=9 | 143.40±40.22 mV/ms n=9 | One-way ANOVA | 0.0011 (***) | 11 DIV |

| **FIGURE 5** |
| --- |

|  | Ctrl (Cav2.1/Cav2.2) | a-syn  (Cav2.1/Cav2.2) | Test statistic | p | DIV |
| --- | --- | --- | --- | --- | --- |
| ΔC | 26.5±12.06 fF n=11 | 88.9±59.32 fF n=15 | t-test | 0.001 (***) | 11 DIV |
| *pC/pF* | 6.4±6 pC/pF n=11 | 22.1±8 pC/pF n=15 | t-test | 0.0011 (***) | 11 DIV |
|  | Ctrl (Cav1/Cav2.3) | a-syn  (Cav1/Cav2.3) |  |  |  |
| ΔC | 38.8±24.2 fF n=8 | 43.5±31.5 fF  n=8 | t-test | 0.738 | 11 DIV |
| *pC/pF* | 22.8±10.26 pC/pF n=8 | 23.5±11.46 pC/pF n=8 | t-test | 0.893 | 11 DIV |

| **FIGURE 6** |
| --- |

|  | Ctrl | *a-syn* | *a-syn+w-MVIIC* | Test statistic | p | DIV |
| --- | --- | --- | --- | --- | --- | --- |
| Frequency | 0.2±0.1 Hz n=7 | 1.9±0.9 Hz n=7 | 0.07±0.04 Hz n=5 | KW-ANOVA | 0.003 (**)  0.009 (**) | 14 DIV |
| Imax | 14.5±7.5 pA n=106 | 23.5±16.8 pA n=2993 | 10.5±5.4 pA n=42 | KW-ANOVA | 0.0011(***)  0.0009(***) | 14 DIV |
| Q | 7.5±5 fC n=106 | 11.1±10 fC  N=2993 | 7±4 fC  N=42 | KW-ANOVA | 0.245  0.091 | 14 DIV |
| t1/2 | 0.33±0.23 ms n=106 | 0.29±0.18 ms  n=2993 | 0.32±0.23 ms  n=42 | KW-ANOVA | 0.634  0.595 | 14 DIV |

| **FIGURE 7** |
| --- |

|  | Ctrl (Cav2.1/Cav2.2) | a-syn  (Cav2.1/Cav2.2) | Test statistic | p | DIV |
| --- | --- | --- | --- | --- | --- |
| pA/pF | 21.1±13.6 pA/pF n=14 | 78.7±41.5 pA/pF n=20 | Mann-Whitney Test | 0.04 (*) | 11 DIV |
|  | Ctrl  (Cav2.1) | a-syn  (Cav2.1) |  |  |  |
| pA/pF | 67.0±20.9 pA/pF n=14 | 67.5±31.4 pA/pF n=18 | Mann-Whitney Test | 0.809 | 11 DIV |
|  | Ctrl  (Cav1) | a-syn  (Cav1) |  |  |  |
| pA/pF | 52.2±25.72 pA/pF n=26 | 45.5±27.15 pA/pF n=18 | Mann-Whitney Test | 0.210 | 11 DIV |
|  | Ctrl  (total) | a-syn  (total) |  |  |  |
| pA/pF | 67.0±21.0 pA/pF n=14 | 62.7±24.6 pA/pF n=17 | Mann-Whitney Test | 0.59 | 11 DIV |

| **FIGURE 8** |
| --- |
| **Hz** |

| ctrl | a-syn | w-MVIIC | sulp | a-syn+w-MVIIC | a-syn+sulp | Test statistic | p | DIV |
| --- | --- | --- | --- | --- | --- | --- | --- | --- |
| 4.6±3.6Hz n=38 | 1.9±2.1 Hz n=50 | 3.6±2.9 Hz n=33 | 4.2±2 Hz n=23 | 4.9±3.4 Hz n=37 | 4.6±3.4 Hz n=23 | Kruskal-Wallis ANOVA | 0.012(***)  0.00012(***)  0.002(**) | 11 DIV |

| **AHP** |
| --- |

| ctrl | a-syn | a-syn+w-MVIIC | a-syn+sulp | Test statistic | p | DIV |
| --- | --- | --- | --- | --- | --- | --- |
| -44.3±5 mV n=37 | -46.7 ±4.42 mV  N=41 | -43.8  ±34.9 mV n=24 | -42.7±4.5 mV n=19 | One-way ANOVA | 0.009(**)  0.012(*)  0.0025(*) | 11 DIV |

| **Tp** |
| --- |

| ctrl | a-syn | a-syn+w-MVIIC | a-syn+sulp | Test statistic | p | DIV |
| --- | --- | --- | --- | --- | --- | --- |
| 6.0±0.4 ms n=37 | 5.8±0.3 ms n=41*;* | 6±3 ms n=24 | 6.1±0.3 ms n=19 | One-way ANOVA | 0.01(***)  0.009(***)  0.038(*) | 11 DIV |

| **dV/dtmax** |
| --- |

| ctrl | a-syn | a-syn+w-MVIIC | a-syn+sulp | Test statistic | p | DIV |
| --- | --- | --- | --- | --- | --- | --- |
| 107±48 mV/ms n=46 | 159±44 mV/ms  n=30 | 100±41 mV/ms n=15 | 87±49 mV/ms n=18 | One-way ANOVA | 0.001(***)  0.00012(***)  0.012(***) | 11 DIV |

| **dV/dt max (50kHz)** |
| --- |

| ctrl | a-syn | a-syn+w-MVIIC | a-syn+sulp | Test statistic | p | DIV |
| --- | --- | --- | --- | --- | --- | --- |
| 75.34±  17.76 mV/ms n=10 | 143.40±  40.22 mV/ms  n=9 | 75.54±  27.71 mV/ms n=7 | 68.12±  23.47 mV/ms n=8 | One-way ANOVA | 1.09E-4(***)  0.0012(***)  0.0011(**) | 11 DIV |
